# Omentum provides a special cell microenvironment for ovarian cancer

**DOI:** 10.1002/cnr2.1858

**Published:** 2023-08-21

**Authors:** Zeying Li, Xiaoling Fang, Sixue Wang

**Affiliations:** ^1^ The Second Xiangya Hospital of Central South University Changsha China

**Keywords:** adipocyte, exercise, metformin, omentum, ovarian cancer

## Abstract

**Background:**

Ovarian cancer seriously threatens women's health because of its poor prognosis and high mortality. Due to the lack of efficient early detection and screening methods, when patients seek doctors' help with complaints of abdominal distension, back pain and other nonspecific signs, the clinical results always hint at the widespread metastasis of disease. When referring to the metastasis of this disease, the omentum always takes precedence.

**Recent findings:**

The distinguishing feature of the omentum is adipose tissue, which satisfies the energy demand of cancer cells and supplies a more aggressive environment for ovarian cancer cells. In this review, we mainly focus on three important cell types: adipocytes, macrophages, and mesenchymal stem cells. Besides, several mechanisms underlying cancer‐associated adipocytes (CAA)‐facilitated ovarian cancer cell development have been revealed, including their capacities for storing lipids and endocrine function, and the release of hormones, growth factors, and adipokines. Blocking the reciprocity among cancer cells and various cells located on the omentum might contribute to ovarian cancer therapy. The inhibition of hormones, growth factors and adipokines produced by adipocytes will be a novel therapeutic strategy. However, a sufficient number of trials has not been performed. In spite of this, the therapeutic potential of metformin and the roles of exercise in ovarian cancer will be worth mentioning.

**Conclusion:**

It is almost impossible to overcome completely ovarian cancer at the moment. What we can do is trying our best to improve these patients' prognoses. In this process, adipocytes may bring promising future for the therapy of ovarian cancer.

## 1 INTRODUCTION

Ovarian cancer has a 1.3% probability of occurrence in women. Although the specific pathogenesis of the disease is poorly elucidated, many results have pointed out that multiple birth history, use of oral contraceptives, avoidance of menopausal hormone use, and ligation of the oviduct reduce the risk of developing ovarian cancer.^1^ Epithelial ovarian cancer (EOC) is the most common type. High‐grade serous carcinoma (HGSC) is the most common epithelial subtype.^2^ Most HGSC patients are diagnosed at stage III or IV, which is consistent with their poor 5‐year survival, compared with other subtypes of ovarian cancer.^3^

The omentum, which is encompassed mainly by adipose tissue, is the site where ovarian cancer is most prone to metastasis. In 1889, Paget first introduced the “seed and soil” principle in terms of cancer metastasis.^4^ Although there are several theories and hypotheses raised to challenge this concept, it is still accepted by majority nowadays.^4^ However, idiographic terms change constantly; for example, “seed” has been renamed cancer stem cells and “soil” has been renamed the tumor microenvironment in most cases.^5^ We are interested in the interaction of the omentum, which acts as “soil,” and ovarian cancer cells, which play the part of “seed.” Although past studies have explained the above mechanisms from various aspects, they either contained only a fraction of the cell species or explored only a fraction of the cytokines, an integrative review has not been performed. Thus, in this review, we provide a systematic overview of these processes in the context of adipose tissue, particularly adipocytes and macrophages that promote the biological behavior of ovarian cancer cells, and discuss the roles of obesity in ovarian cancer from an overall perspective. It is obvious that a comprehensive understanding of the above constants is necessary for clinical and basic research.

## 2 VARIOUS CELL COMPONENTS IN THE OMENTUM PLAY ROLES IN OVARIAN CANCER DEVELOPMENT

**FIGURE 1:**
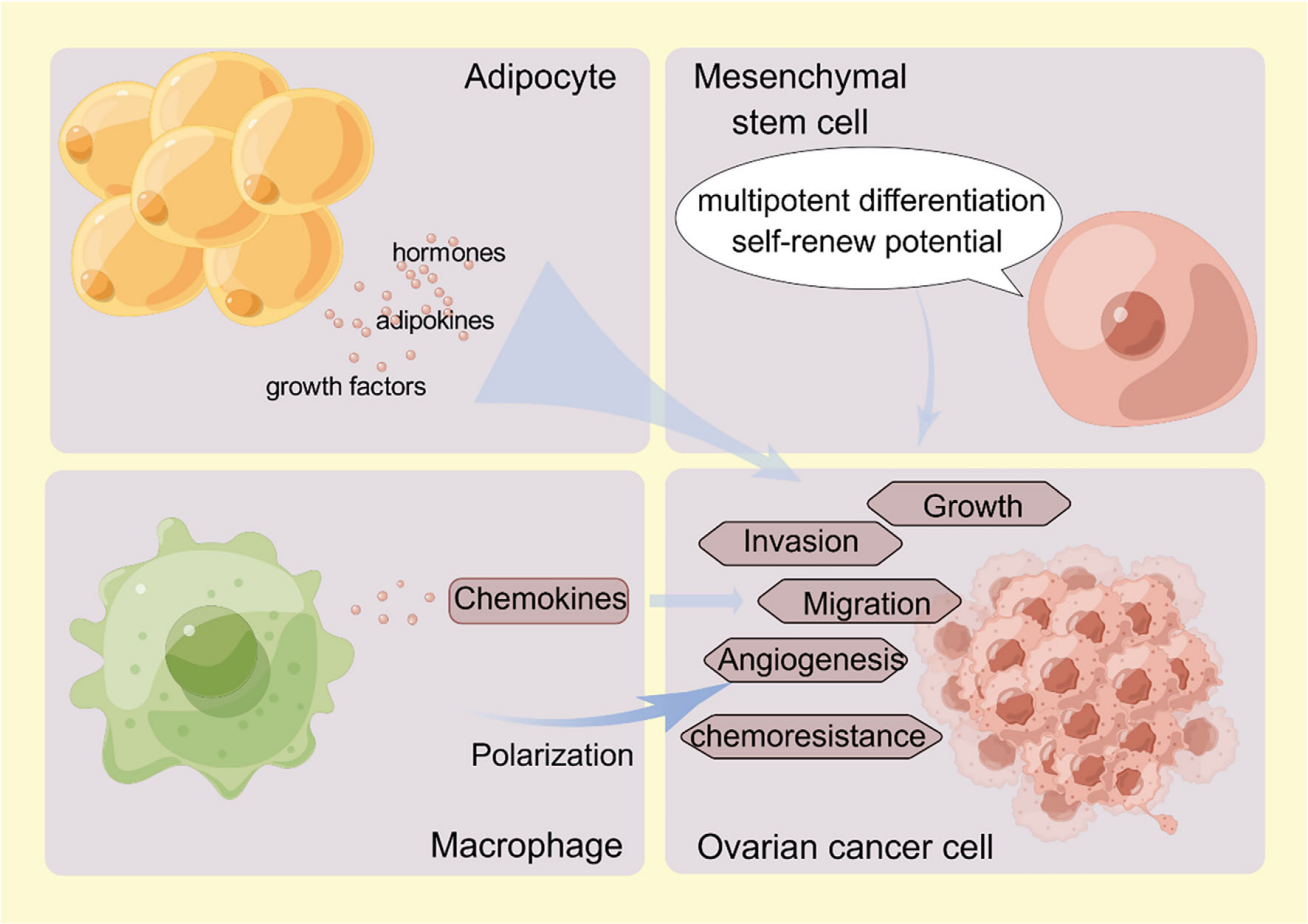
In the microenvironment of adipose tissue infiltration, various cell components support adipose tissue and play important roles in ovarian cancer development via their specific functions and unique characteristics. Adipocytes, as the dominant cells in this environment, can secrete many kinds of cytokines and factors to enhance ovarian cancer cell growth, invasion, migration, angiogenesis and chemoresistance. Macrophages and mesenchymal stem cells depend on their own plasticity to make the local environment more suitable for ovarian cancer cells. By Figdraw.

Wilkosz et al.^6^ concluded that the human greater omentum is composed of an adipose‐rich region and is translucent and membranous by means of phase contrast microscopy, scanning electron microscopy (SEM), and transmission electron microscopy (TEM). The former contains a great deal of milky spots, a cluster of stromal cells and immune cells, including B cells, T cells, NK cells, macrophages, and so forth, which are also named fat‐associated lymphoid clusters. Clark et al.^7^ proposed a two‐step model to clarify the roles of milky spots and adipocytes: Milky spots participate in the location of ovarian cancer cells, and adipocytes play part in the subsequent migration and invasion. Neither T cells nor B cells can assist ovarian cancer cell infiltration, however, macrophages play opposing roles. Mesenchymal stem cells can be found widely in various tissues. They can boost the progression and metastasis of ovarian cancer by their multipotent differentiation ability, self‐renewal potential, immunomodulatory, and secretion function.^8^ Remarkably, mesenchymal stromal cells (MSCs) deprived of omentum adipose tissue show distinctive characteristics when compared with mesenchymal stromal cells deprived of adipose tissue from other sites. Existing experimental results have demonstrated that adipose‐derived mesenchymal stem cells would enhance the growth, migration, and invasion.^9^, ^10^ Therefore, the concrete roles and mechanisms of adipocytes, macrophages, and stromal cells in ovarian cancer will be discussed below. Figure 1 has shown the relationship among these cells and ovarian cancer.

### 2.1 Cancer‐associated adipocytes deprived of omentum

Cancer‐associated adipocytes (CAAs) might directly influence ovarian cancer cell malignant behaviors by infiltrating into tumor cells, other adipocytes are located around cancer cells and influence cancer cells indirectly. And a cluster of adipocytes remodeled by tumor cells have roles akin to magic.^11^

Generally, we have reached a consensus that the oxidative metabolism deregulated in cancer cells. They tend to utilize glycolysis to produce energy, which is different from healthy cells. This characteristic is named the Warburg effect.^12^ Ovarian cancer cells are no exception. However, while coculturing adipocytes and ovarian cancer cells, the alterations in lipid metabolism in ovarian cancer cells deserve attention. Regardless of whether adipocytes are cocultured with ovarian cancer cell lines in vitro or cancer cells adjacent to the omentum in vivo, an increase in lipid peroxidation in ovarian cancer cells to meet their surge in energy demand is observed, and this process mainly depends on adipocytes, which act as a “lipid library.” Some factors produced or regulated by adipocytes also support cancer cell lipid metabolism reprogramming from lipid synthesis to catabolism. For example, mass spectrometry of the proteins regulated by coculturing with adipocytes and a comparison of data for primary and metastatic tumors from a public dataset revealed the same changes, in which CD36, FABP4, and ADH‐1 were significantly upregulated under the influence of adipocytes.^13^, ^14^

The complex and vital functions and mechanisms involved in modulating ovarian cancer cell growth and progression will be summarized. In this part, we mainly refer to some hormones, adipokines and other factors that are released or associated with adipocytes. And the roles of these factors are summarized in the Table 1.

**TABLE 1:** Examples of adipocyte‐associated factors.

Factors	Functions (for ovarian cancer cells)	References
Leptin	Growth; invasion	17, 18, 19, 20, 21, 22
Resistin	Angiogenesis	23, 24, 26
Wnt5a	Adhesion; migration; metastasis	26, 27
MCP‐1	Invasion; migration; metastasis	28, 29
FABP‐4	Growth; metastasis; angiogenesis; lipid accumulation	31, 32, 33, 34
CD36	Proliferation; invasion; migration; lipid uptake	35, 36, 37
ADH‐1	Proliferation; invasion; migration; carcinogenic effects	38, 39, 40
SIK‐2	Growth; metastasis; fatty acid oxidation	41, 42, 43
DPYSL4	Energy metabolism; poor survival	44, 45, 46
miR‐21	Proliferation; invasion; chemosensitivity	47, 48, 49
Bcl_xl_	Chemosensitivity	50, 51, 52

#### 2.1.1 Leptin and leptin to adiponectin (L:A) ratio

Leptin is a kind of adipocyte‐secreted hormone and plays different roles in ovarian cancer. It can promote ovarian cancer cell growth through cyclin D1, a cancer cell growth sensor, and Mcl‐1, an anti‐apoptotic factor.^15^ The expression of uPA induced by leptin mediates ovarian cancer cell invasion.^16^ Flow cytometry results have verified that leptin is associated with chemoresistance of ovarian cancer.^17^ Several particular mechanisms are involved in the above roles, including the MEK/ERK1/2 pathway, PI3K̸Akt pathway, RhoA/ROCK pathway, estrogen receptor pathway,^18^ and so forth. But there are some opposite conclusions. The molecule alone has no obvious effect on ovarian cancer. It is interesting that Słomian^19^ combined leptin and adiponectin and used their ratio as indicator of the response to chemotherapy. Adiponectin is another adipokine produced by adipocytes. It acts different roles in various cells. For example, it can take part in the cell differentiation and regulate the endocrine function of adipose tissue.^20^ There is a mountain of evidence which suggests that this factor has anti‐carcinogenic effects.^21^ Some agents which can increase the level or stimulate the activity of adiponectin would be hopeful for the therapy of ovarian cancer.^22^ In fact, this has given us a meaningful tip. Besides exploring the roles of various adipocytokines, the interaction among these factors is also necessary.

#### 2.1.2 Resistin

Resistin is a novel adipocytokine that is secreted by human adipocytes and mononuclear cells.^23^ The exiting results have revealed that the higher level of resistin, the poorer prognosis of ovarian cancer. It can enhance the angiogenesis process, epithelial‐mesenchymal transition and stemness of ovarian cancer cells.^24^ Recombinant human resistin enhanced the expression of VEGF in a time‐ and dose‐dependent manner in human ovarian cancer cell lines. The PI3K/Akt‐Sp1 pathway mediates the above effects of resistin. However, additional in vivo studies on the functional network among these factors are lacking.^25^

#### 2.1.3 Wnt5a

Wnt5a is a highly evolutionarily conserved noncanonical Wnt ligand^26^ that is involved in ovarian cancer metastasis. It is mainly produced by peritoneal mesothelial cells and visceral adipose tissue. In ex vivo experiments, ovarian cancer cell lines acquire greater adhesion and migration ability under the influence of recombinant wnt5a. WNT5A knockout mice achieved by crossing WNT5A‐floxed mice (Wnt5afl/fl) with UBC‐Cre/ERT2 mice were distinguished from the control tumor group at the cytokine level, including cytokines that regulate immune cell chemotaxis. Practically speaking, knocking out WNT5A will contribute to a higher CD8+/−FOXP3+ ratio and M1/M2 macrophage ratio, and both of them indicate better disease prognosis. Further studies show that the Src family kinase Fgr is its downstream effector. Some selective inhibition of Fgr kinase activity might be exerted to treat ovarian cancer.^27^

#### 2.1.4 MCP‐1

Monocyte chemotactic protein‐1 (MCP‐1) is also known as chemokine (C‐C motif) ligand 2 (CCL‐2).^28^ MCP‐1 produced by omental adipocytes can bind to its receptor CCR‐2 to regulate the expression of VEGF‐A via the PI3K/AKT/mTOR pathway. In vitro migration and invasion assays, this axis also helped ovarian cancer cells gain more aggressive characteristics. Either MCP‐1 neutralization antibody or CCR‐2 antagonist could weaken the effects.^29^

#### 2.1.5 FABP4

FABP4 is mainly produced by adipocytes and macrophages and participates in the regulation of intracellular lipids by binding and redistributing them normally.^30^ However, adipocyte‐induced FABP4 expression can promote ovarian cancer cell proliferation and metastasis both in vivo and in vitro. The inhibition of FABP4 by CRISPR and siRNA reduced the capacity of adipocyte cocultured ovarian cancer cells to accumulated lipids, and with the impact of this, adipocyte‐relevant β‐oxidation, ROS generation and lipid peroxidation were affected. It often increases ATP‐production by glycolysis and reduces mitochondrial ATP production. Some addition of glycolysis process products might cycle arrest. Harjes et al.^31^ discovered that silencing FABP through siRNA contributes to the inhibition of angiogenesis, growth and metastasis in vivo. Furthermore, this factor can be regulated by some cytokines. For example, IL‐17A, a vital proinflammatory cytokine, has been found to upregulate FABP4 to realize more fatty acid uptake through the IL‐17A/IL‐17RA/p‐STAT3/FABP4 axis to help ovarian cancer cell growth and metastasis in an adipose‐rich environment.^32^ Therefore, some molecular inhibitors targeting FABP4 might block its function and bring a promising future for ovarian cancer therapy, such as BMS309403, which was initially used to treat atherosclerosis and type 2 diabetes and has been proven to increase platinum‐based drug sensitivity in vivo.^13^, ^33^, ^34^

#### 2.1.6 CD36

Adipocytes from human greater omentum can induce the expression of CD36 in ovarian cancer cells, which is a unique feature distinguishing it from other omental cell types, including fibroblasts and macrophages. The expression of CD36 can increase the uptake of fatty acids and lipid accumulation, as measured by fluorescently labeled fatty acid analogs and immunofluorescent staining for neutral lipids. The inhibition of this factor would cripple its abovementioned roles. At the same time, the results of gene expression analysis demonstrated the downregulation of acetyl‐CoA carboxylase (ACACA), the rate‐limiting enzyme in FA synthesis.^35^ Transcription factor analysis revealed that several lipogenic genes were also downregulated, such as Sterol Regulatory Element Binding Transcription Factors (SREBPF1 and SREBPF2). Sterol regulatory element binding proteins (SREBPs) are the most important transcription factors in lipid homeostasis. It has three isoforms, SREBP‐1a, SREBP‐1c, and SREBP‐2. SREBP‐1c mainly regulates fatty acid synthesis, and SREBP‐2 is specifically involved in cholesterol synthesis.^36^ All these facts indicate that omental adipocytes can alter ovarian cancer metabolism by CD36; they can not only promote exogenous lipid uptake rather than endogenous lipid synthesis but also enhance anaerobic glucose metabolism while suppressing glucose oxidation. Furthermore, in vitro experiments, CyQuant cell proliferation assays and transwell assays showed that CD36 can promote ovarian cancer cell proliferation, invasion and migration. SKOV3ip1 and OVCAR8 xenograft mouse models also indicate that CD36 regulates the metastasis of ovarian cancer.^37^

#### 2.1.7 ADH‐1B

Analyses of data from public datasets have shown that ADH‐1B (alcohol dehydrogenase 1B) is one of the candidates for forecasting residual ovarian cancer.^38^ It can fuel the progression and infiltration of ovarian cancer cells in vivo and in vitro. ADH‐1B mainly mediates ethanol conversion to acetaldehyde. Therefore, with the upregulation of ADH‐1B, acetaldehyde may accumulate gradually.^39^ In fact, acetaldehyde is toxic to cells, has carcinogenic effects, and disrupts the DNA repair and methylation processes.^40^ However, specific and systemic studies on ADH‐1B in ovarian cancer still exhibit a large gap.

#### 2.1.8 SIK‐2

Dysregulation of fatty acid and cholesterol synthesis plays an important role in ovarian cancer. Salt‐inducible kinase 2 (SIK2) is overexpressed in adipocyte‐rich metastases^41^ and can enhance the expression of FASN (one of the rapid‐limiting enzymes in fatty acid synthesis) and HMGCR (one of key enzymes in cholesterol synthesis) to promote ovarian cancer cell multiplication and metastasis in vitro and in vivo.^42^ Adipocytes can activate SIK‐2 autophosphorylation through the Ca^2+^ pathway. SIK‐2 can participate in fatty acid oxidation and mitochondrial respiration, which might sustain adipocyte‐induced metastasis of ovarian cancer.^41^ Furthermore, SIK‐2 can also directly phosphorylate MYLK and activate its downstream pathway to boost ovarian cancer cell motility.^43^

#### 2.1.9 DPYSL4

RNA sequencing and chromatin immunoprecipitation (ChIP)‐sequence analyses have shown that dihydropyrimidinase‐like 4 (DPYSL4) is a regulator of downstream of P53. Metabolome analysis verified higher concentrations of glycolysis intermediates in HCT116 human non‐small cell lung cancer cells without P53 expression, in accordance with tumor cells preferentially using glycolysis rather than OXPHOS to meet their rapid energy demand. 2D Blue Native SDS polyacrylamide gel electrophoresis (BN/SDS/PAGE) was used to confirm that DPYSL4 is associated with mitochondrial supercomplexes I, III, and IV. The oxygen consumption rate (OCR) and the NAD+/NADH ratio also indicate that DPYSL4 plays roles in mitochondrial respiration, which rescues the Warburg effect in cancer cells. The function of DPYSL4 in tumor cell energy metabolism provides a novel angle of view for antitumor metabolism. For ovarian cancer, Kaplan–Meier survival analyses have shown that DPYSL4 is associated with poor survival in ovarian cancer.^44^, ^45^ Unfortunately, this factor lacks further insightful investigations in ovarian cancer.^46^

#### 2.1.10 miR‐21

Next‐generation sequencing has revealed that RNA expression is different in exosomes isolated from ovarian cancer cells and adipocytes and fibroblasts from normal human omental tissue and cancer‐associated omental tissue. MiR‐21 is the most abundant.^47^ Though influencing the activity of PI3K/AKT mediated by PTEN, the upregulation of miR‐21 will promote ovarian cancer cell proliferation and inhibit cancer cell apoptosis.^48^ In addition, it is involved in the chemoresistance progress by CD44v6 pathway.^49^ There are still many research gaps remaining about its potential roles in ovarian cancer.

#### 2.1.11 Bcl_xl_

Alvero et al.^50^ regarded CD44+/MyD88+ epithelial ovarian cancer (EOC) stem cells as a chemoresistance phenotype. The Bcl2 family members show evident variation in chemoresistance models and can determine cancer cell survival or apoptosis. Gene expression microarray analysis revealed that BCL2L1 is the most differentially expressed gene in chemotherapy‐resistant ovarian cancer cells compared with chemotherapy‐sensitive ovarian cancer cells, and the western blot results also prove that Bcl_xl_, encoded by BCL2L1, is differentially expressed. On the other hand, the adipocyte‐infiltrated microenvironment always upregulates the expression of Bcl_xl_. Bclxl‐specific siRNA will achieve apoptosis of chemoresistant ovarian cancer cells.^51^, ^52^

### 2.2 Cancer‐associated macrophages deprived from the omentum

In recent years, increasing attention has been focused on cancer‐associated macrophages (CAMs). Macrophages have a variety of effects on the basis of their extreme plasticity in response to their microenvironment.^53^ In many previous studies, most foci are gathered in macrophages isolated from peritoneal ascites because they are abundant in thoracic and ascites of ovarian cancer patients. Relevant results have demonstrated that ascites‐deprived macrophages express M1 and M2 polarization markers, which is named mixed polarization.^54^ In this review, we will discuss reciprocity between ovarian cancer cells and macrophages deprived from omentum adipose tissue. Unfortunately, relevant studies are limited. Thus, whether previous results about the roles of macrophages stemming from peritoneal ascites apply to macrophages originating from omentum adipose tissue should be further verified.

Peritoneal injection into mice of the immortalized mouse ovarian epithelial cell line ID8, which is labeled with Qdots (Qtracker705), demonstrated that the area of aggregated omental macrophages attracts more ovarian cancer cells. Further single‐cell RNA sequencing analysis of these macrophages indicated that common coexpressing macrophage markers are Lyve‐1, Cd163, and Tim4. Only CD169^hi^ macrophages expressed Lyve‐1 at the same time, and only CD169^hi^ Lyve‐1^+^ cells expressed Cd163 and Tim4. According to their different expression status, the authors divided four subtypes: CD163^+^ Tim^4+^ (P1), CD163^+^ Tim4^−^ (P2), CD163^−^ Tim4^−^ (P3), and CD163^−^ Tim4^+^. The significant feature of P1 macrophages, which are distinguished from other subtypes, is they might originate from embryos rather than monocytes, which is why they cannot be replaced easily by other cell types. The specific deletion of P1 macrophages would inhibit ovarian cancer cell metastasis, and their existence would be the basis of the acquisition of invasive behavior of ovarian cancer cells.^55^

In addition, chemokines and their receptors form an important bridge between macrophages and cancer cells. For example, in mouse models, the expression of the chemokine ligand CCL6 presents significant changes in omental macrophages while ovarian cancer cells colonize the omentum. Similarly, CCL23, the human homolog of CCL6, was discovered in human omental macrophages. CCR1, which is highly expressed in ovarian cancer cells, can mediate ovarian cancer cell‐enhanced migration and metastasis.^56^ Some blockers targeting at CCR1 or CCL23 might improve the clinical outcome of ovarian cancer patients.

### 2.3 Cancer‐associated mesenchymal stem cells deprived of omentum

In recent years, mesenchymal stem cells (MSCs) have attracted attention because of their therapeutic potential in cancer. Tang et al.^57^ found that MSCs deprived of omentum adipose tissue tend to express more carcinoma‐associated‐fibroblast markers through the TGF‐β pathway to support ovarian cancer cell growth and omental metastasis. Inhibition of this differentiation would be a new therapeutic target. Coculturing ovarian cancer cell lines and adipose‐derived mesenchymal stem cells (ADSCs) in vitro and in mouse xenograft models reached the same conclusion: adipose‐derived mesenchymal stem cells could promote ovarian cancer cell growth and migration, and above process was inhibited by downregulating the expression of MMP.^58^ Other phenotype validation experiments illustrated that MSCs deprived of omentum adipose tissue would advance invasion and chemoresistance.^59^ There is another perspective that ADSC‐deprived exosomes could induce the apoptosis of ovarian cancer cells, and the sequencing results showed a great deal of miRNAs associated with ovarian cancer survival.^60^ Each conclusion provides an important disease treatment idea for ovarian cancer.

## 3 PREVENTION AND THERAPY

Because of the lack of early detection and acquisition of drug resistance, complete victory over ovarian cancer is still extremely difficult. The current mainstream treatment includes primary debulking surgery (PDS) combined or not combined with chemotherapy or interval debulking surgery (IDS) combined with neoadjuvant chemotherapy. When patients finish primary therapy and achieve a complete clinical response or partial response, maintenance therapy is administered to improve their progression‐free survival (PFS) and overall survival (OS).^61^, ^62^ To unearth more latent mechanisms is significant. It is evident that omentum can provide a suitable environment for ovarian cancer cells. From the perspective of the roles of adipose cells and adipocytes, metformin might be promising for ovarian cancer therapy. More epidemiologic evidence supports that exercise could reduce ovarian cancer risk.^63^

### 3.1 Metformin

Metformin is a classical hypoglycemic drug that could also be effective in several cancers, including ovarian cancer. It can inhibit the conversion of preadipocytes to adipocytes and block the biological process of adipocytes to interfere with ovarian cancer cell growth and invasion mediated by adipocytes.^64^ Relevant clinical trials have proven that metformin is associated with better prognosis in ovarian cancer patients. It can downregulate the activity of IL‐6/STAT3 and influence the expression of VEGF and TGF‐β1. It can enhance sensitivity to cisplatin in ovarian cancer by altering the methylation of cancer‐stem cells.^65^, ^66^ Currently, a nonrandomized phase II study combining metformin and chemotherapy in advanced‐stage ovarian cancer without diabetes has reached promising conclusions.

### 3.2 Exercise and weight control

Some systematic reviews have concluded that exercise is always relevant to better outcomes of ovarian cancer.^67^ Appropriate exercise is helpful for weight control, and increasing evidence supports that obesity, particularly the stock of visceral white adipose tissue (WAT), increases the occurrence and mortality of ovarian cancer.^68^ The classical action mechanism is concentrated on secretion of adipokines, insulin resistance, and chronic inflammation.^69^, ^70^ Liu et al.^71^ confirmed that obesity and a high‐fat diet influence immune cell infiltration. For example, CD45^+^ lymphocyte (B‐cell marker), whole macrophage, and M1‐polarized macrophage infiltration decreased in the obesity group while M2‐polarized macrophages showed no significant change. The alteration of the immune microenvironment might open up another mechanism linking obesity and ovarian cancer. Recent data have suggested that exercise might contribute to the activation of M1 macrophages, which arouse antitumor immune responses.^72^ Exercise intervention during or following ovarian cancer therapy might improve the lives of these patients.

## 4 CONCLUSIONS

The omentum acts as the most frequent location where ovarian cancer cells spread for subsequent metastasis. It covers the surface of abdominal organs, and its enormous endocrine function and particular structural composition can provide a suitable and specific environment for ovarian cancer cell growth, invasion, migration and chemoresistance. Various cell compositions take part in ovarian cancer genesis and development via energy metabolism regulation, immune reactions and many other processes. All of the above molecules are just the tip of the iceberg. Investigations performed to reveal additional functions of the omentum, especially adipose tissue, might offer more effective and satisfactory therapeutic solutions for patients suffering from ovarian cancer. Further study of the roles of the tumor cell microenvironment in ovarian cancer will facilitate its immunotherapy. Some agents which targeted for the above cytokines will be hopeful for prognosis of ovarian cancer.

## AUTHOR CONTRIBUTIONS

**Zeying Li:** Visualization (lead); writing – original draft (lead). **Xiaoling Fang:** Writing – review and editing (lead). **Sixue Wang:** Visualization (supporting); writing – review and editing (supporting).

## CONFLICT OF INTEREST STATEMENT

The authors have stated explicitly that there are no conflicts of interest in connection with this article.

## Data Availability

Data openly available in a public repository.
